# Emotions and Incivility in Vaccine Mandate Discourse: Natural Language Processing Insights

**DOI:** 10.2196/37635

**Published:** 2022-09-13

**Authors:** Hannah Stevens, Muhammad Ehab Rasul, Yoo Jung Oh

**Affiliations:** 1 University of California, Davis Davis, CA United States

**Keywords:** vaccine hesitancy, COVID-19, vaccine mandates, natural language processing, incivility, LIWC, Linguistic Inquiry and Word Count, Twitter

## Abstract

**Background:**

Despite vaccine availability, vaccine hesitancy has inhibited public health officials’ efforts to mitigate the COVID-19 pandemic in the United States. Although some US elected officials have responded by issuing vaccine mandates, others have amplified vaccine hesitancy by broadcasting messages that minimize vaccine efficacy. The politically polarized nature of COVID-19 information on social media has given rise to incivility, wherein health attitudes often hinge more on political ideology than science.

**Objective:**

To the best of our knowledge, incivility has not been studied in the context of discourse regarding COVID-19 vaccines and mandates. Specifically, there is little focus on the psychological processes that elicit uncivil vaccine discourse and behaviors. Thus, we investigated 3 psychological processes theorized to predict discourse incivility—namely, anxiety, anger, and sadness.

**Methods:**

We used 2 different natural language processing approaches: (1) the Linguistic Inquiry and Word Count computational tool and (2) the Google Perspective application programming interface (API) to analyze a data set of 8014 tweets containing terms related to COVID-19 vaccine mandates from September 14, 2021, to October 1, 2021. To collect the tweets, we used the Twitter API Tweet Downloader Tool (version 2). Subsequently, we filtered through a data set of 375,000 vaccine-related tweets using keywords to extract tweets explicitly focused on vaccine mandates. We relied on the Linguistic Inquiry and Word Count computational tool to measure the valence of linguistic anger, sadness, and anxiety in the tweets. To measure dimensions of post incivility, we used the Google Perspective API.

**Results:**

This study resolved discrepant operationalizations of incivility by introducing incivility as a multifaceted construct and explored the distinct emotional processes underlying 5 dimensions of discourse incivility. The findings revealed that 3 types of emotions—anxiety, anger, and sadness—were uniquely associated with dimensions of incivility (eg, toxicity, severe toxicity, insult, profanity, threat, and identity attacks). Specifically, the results showed that anger was significantly positively associated with all dimensions of incivility (all *P*<.001), whereas sadness was significantly positively related to threat (*P*=.04). Conversely, anxiety was significantly negatively associated with identity attack (*P*=.03) and profanity (*P*=.02).

**Conclusions:**

The results suggest that our multidimensional approach to incivility is a promising alternative to understanding and intervening in the psychological processes underlying uncivil vaccine discourse. Understanding specific emotions that can increase or decrease incivility such as anxiety, anger, and sadness can enable researchers and public health professionals to develop effective interventions against uncivil vaccine discourse. Given the need for real-time monitoring and automated responses to the spread of health information and misinformation on the web, social media platforms can harness the Google Perspective API to offer users immediate, automated feedback when it detects that a comment is uncivil.

## Introduction

### Background

The emergence of the novel coronavirus (COVID-19) has caused 5,878,328 confirmed deaths worldwide as of February 2022, along with 423,437,674 confirmed infections [1]. Despite vaccine availability, vaccine hesitancy has inhibited public health officials’ efforts to mitigate the COVID-19 pandemic, especially in the United States [2]. Although some US elected officials have responded by issuing vaccine mandates, others have amplified vaccine hesitancy by broadcasting messages that minimize vaccine efficacy [3,4].

With 68% of American adults reporting social media as a source of their news diet [5], social media platforms such as Twitter have become important communication channels for US politicians to share their agendas [6]. As a result, social media have become a prominent source of political information and misinformation, including information surrounding COVID-19 vaccines [7-11]. The politically polarized nature of COVID-19 information on social media has given rise to an infodemic, wherein health attitudes often hinge more on political ideology than science [12-15]. As a result, political affiliation influences negative sentiment toward the vaccine [16]. Such negative sentiment may foster uncivil discourse toward the vaccines and mandates [17,18].

Incivility on social media platforms has been widely studied and discussed in both political and health contexts, among others [19-25]. However, to the best of our knowledge, incivility has not been studied in the context of discourse regarding COVID-19 vaccines and mandates. Specifically, there is little focus on the psychological processes that elicit uncivil vaccine discourse. We aimed to bridge this gap by conducting a computational analysis of tweets. In this study, we investigated the role of negative emotion in predicting uncivil posts about COVID-19 vaccine mandates on Twitter. Ultimately, we argue that a more nuanced understanding of the psychological processes underlying uncivil vaccine discourse has practical implications for public health interventions.

### The Role of Negative Emotion in Vaccine Mandate Incivility

Incivility has become a salient point of discussion in social media research. However, scholars across fields have found it difficult to conceptualize incivility. Incivility has been defined in a variety of ways, including impoliteness, profanity, and specific discriminatory acts (eg, former US president Trump caught on a hot mic in 2016 praising nonconsensual sexual encounters with women) [26-29]. Papacharissi [29] supplements this definition by including threat—in this case to democracy—as uncivil. Other scholars have operationalized incivility as including the use of all capital letters, accusations of lying, pejorative speech, ideologically extreme language, exaggerated argument, and misinformation [26,30-33]. Despite these inconsistent operationalizations, incivility is a concept that is nuanced and varies across individuals, perhaps because it is bound by cultural perceptions and understandings of what uncivil discourse is [16,18]. Informed by the operational inconsistency of incivility outlined in the literature, we conceptualize incivility as a multifaceted construct encompassing a diversity of uncivil behaviors, including toxicity, severe toxicity, profanity, threats, insults, and identity attacks in discourse. Recent studies have argued that uncivil behaviors are related to toxicity on social media platforms [34]. Tromble [28] asserts that profanity and insulting language constitute key indicators of uncivil behaviors. Likewise, scholars have argued that identity attacks and threatening language that aims to morally attack individuals or groups are also aspects of incivility and uncivil discourse [35]. We now shift our attention to explaining what causes incivility.

Incivility does not have a single cause; instead, varying forms of uncivil behaviors are a result of diverse psychological processes. For example, a user may post profane content because they are anxious, whereas a user might make an insulting comment because they are angry. However, scholars often obscure these distinct underlying psychological mechanisms by conceptualizing incivility as a one-dimensional process with a unitary explanation [19,21]. In the context of COVID-19 vaccines and mandates, emotional responses such as anger and anxiety among other negative emotions are salient in the discourse about the pandemic [36,37]. In fact, studies have found negative emotions such as anger and anxiety to play a role in driving vaccine hesitancy [38]. We investigated 3 psychological processes that are likely to predict discourse incivility—namely, anxiety, anger, and sadness.

#### Anxiety and Incivility

Anxiety about the safety of the COVID-19 vaccine, paired with dismissive attitudes toward COVID-19’s threat, has a sizable segment of the United States indicating their unwillingness to get vaccinated [38-40]. In line with extant theory asserting that fear-based aggression is the most prevalent when a perceived threat is inescapable [41-43], a fear of harm from the vaccine, as perpetuated by elected officials and media alike, is often followed by avoidance strategies (eg, refusing the vaccine) [9-11,44]. Accordingly, policies that mandate the hesitant to get vaccinated inhibit the ability to escape the threat, and as a result, individuals may react with incivility. Indeed, stress and anxiety have been demonstrated to predict a wealth of uncivil behaviors, including cyber aggression and bullying during COVID-19 [45-47]. Thus, we posit the following.

Hypothesis (H) 1: Anxiety will positively predict post incivility.

#### Anger and Incivility

COVID-19 vaccine mandates have drawn the ire of segments of the United States, including political elites and media outlets who have fueled public outrage about the threat to personal freedoms that vaccine mandates impose [48,49]. Simultaneously, the lack of confidence in vaccine safety and efficacy has segments of the population feeling threatened by the health risks they perceive to be associated with the vaccine. Anger can be understood as an adaptive response to a threat [44]; indeed, a study by Featherstone and Zhang [44] found vaccine misinformation to negatively impact attitudes toward vaccines through anger. Although anger has the functional value of suppressing fear and potentiating a sense of personal control in the face of threat, it can also propel uncivil behavior, including acts of aggression and dismissiveness directed toward those with opposing views [50-52]. Thus, we can expect anger to foster incivility in COVID-19 vaccine mandate discourse.

H2: Anger will positively predict incivility.

#### Sadness and Incivility

Feelings of sadness have been linked with uncivil behavior, including acts of cyber aggression [47,53]. The freedom to travel, remain employed, socialize in groups, eat in restaurants, go to the gym, and more is increasingly determined by one’s vaccination status [54,55]. Thus, mandates that prohibit the unvaccinated from participating in the relationships and activities available to those who are vaccinated may exacerbate existing sadness and depression induced by preexisting COVID-19 lifestyle disruptors [56,57]. Furthermore, social exclusion can elicit sadness and feelings that a group (ie, the unvaccinated) has experienced wrongs that must be righted—a mindset political scientists have coined “victimhood” [58]. Victimhood mentality may prompt individuals to retaliate against vaccine mandates and manifest as uncivil behaviors. Accordingly, we predict the following.

H3: Sadness will positively predict incivility.

## Methods

### Data Collection

**Figure 1:**
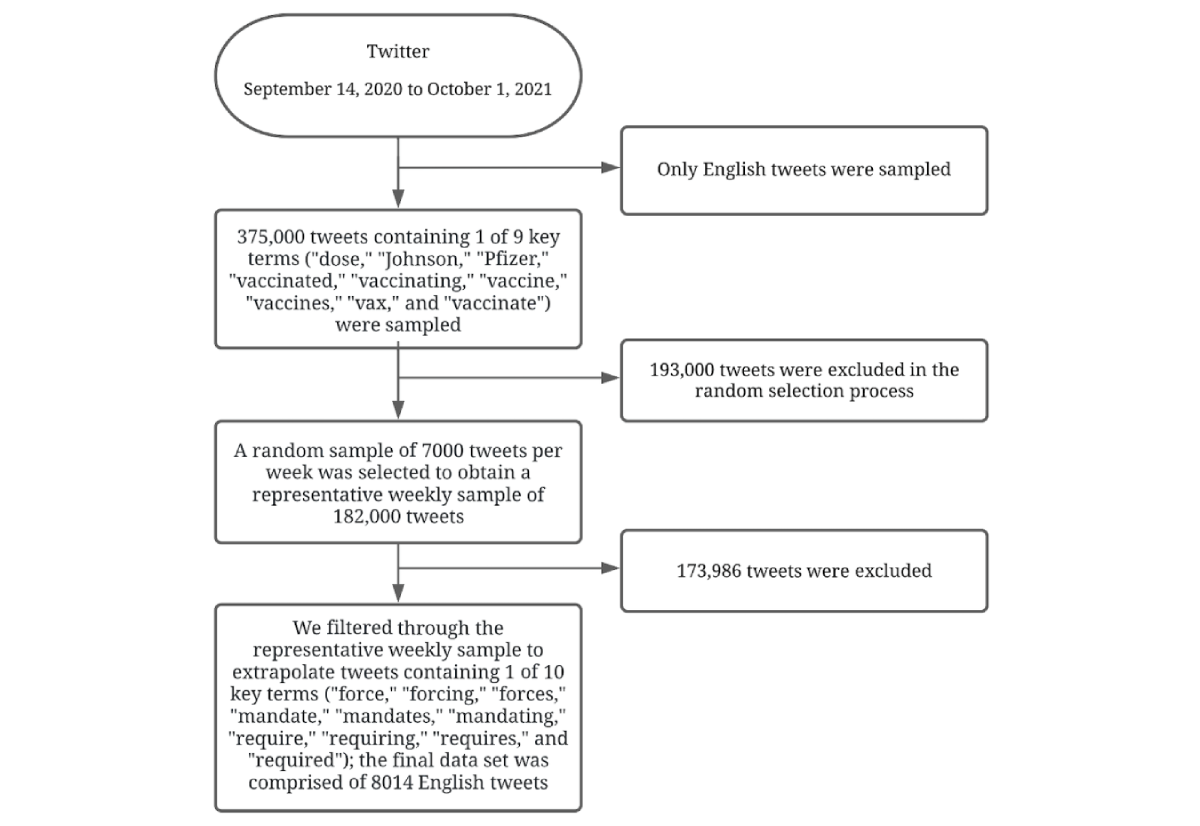
Flowchart of the data collection process.

The sample comprised posts shared to Twitter, a popular platform for seeking and sharing health information on the web, including (mis)information about vaccination and vaccines [7-11]. We opted to curate a list of vaccine-related words and scraped tweets containing those words. We curated a list of words that we believed would collect tweets related to the vaccine, without introducing bias into the data set. For example, “shot” was not included, because we noticed that it scraped tweets about gunshots, which are unrelated to the COVID-19 vaccine. The text of the 8014 tweets contained terms related to COVID-19 vaccine mandates (eg, “Moderna,” “required,” and “mandating”) from September 14, 2020, to October 1, 2021. See Figure 1 for a flowchart of the data collection process.

Twitter’s code-free application programming interface (API) Tweet Downloader Tool (version 2) was used to extract posts about COVID-19 vaccine mandates. We were interested in words that would identify tweets about COVID-19 vaccine mandates rather than the COVID-19 vaccine generally. Thus, we filtered through a data set of 375,000 vaccine-related tweets posted from September 14, 2020, to October 1, 2021, to extrapolate tweets specifically related to vaccine mandates (eg, “forcing,” “required,” and “mandating”) from September 14, 2020, to October 1, 2021; the final sample contained 8014 tweets.

### Natural Language Processing Procedures

The data were analyzed using 2 different natural language processing approaches: (1) the Linguistic Inquiry and Word Count (LIWC) computational tool [59] and (2) the Google Perspective API [60].

#### LIWC Sentiment Analysis

LIWC is a natural language processing tool that measures psychological processes in texts by counting the percentage of words in a given tweet that fall into prespecified categories. It has been validated and used in investigations of mental health during the COVID-19 pandemic (eg, LGBTQ+ youth mental health) [12,61]. In contrast to other sentiment analysis lexicons that generate the valence of emotion (eg, Afinn and Bing, which assign texts a score from negative to positive) without extrapolating discrete emotions and sentiment analysis lexicons that produce binary outcomes (eg, NRC), we wanted a continuous measure of the extent to which texts had a particular sentiment [62]. Although there are multiple tools that continuously capture sentiment and emotions using natural language processing methods (eg, IBM Watson) [63], we specifically used the LIWC dictionary for emotion classification, because compared to the aforementioned natural language processing tools, the LIWC dictionary has been validated in multiple studies, and thus, we considered that it would present a more accurate estimate of the level of emotions reflected in the textual data. We leveraged LIWC to measure the valence of linguistic anger (eg, “frustrated,” and “annoyed”), sadness (eg, “hopeless,” and “miserable”), and anxiety (eg, “afraid,” and “stressed”) in texts [59]. Tweets had an average anxiety score of 0.79 (SD 1.67), an average anger score of 0.11 (SD 0.75) and an average sadness score of 0.09 (SD 0.52).

#### Google Perspective API Machine Learning Analysis

To measure dimensions of post incivility, we used the Google Perspective API to measure levels of toxicity, severe toxicity, insult, profanity, threat, and identity attacks in tweets related to vaccine mandates (see Table 1) [60]. The Google Perspective API is a tool designed by Google’s Counter-Abuse Technology Team that measures incivility in web-based posts.

The Google Perspective API model is trained by human coders on a data set of millions of comments from a variety of web-based sources, including forums (eg, Wikipedia). The model is robust and has been used in a variety of contexts, from political incivility to rape culture to COVID-19 vaccine information [21,64,65]. For example, Hopp et al [64] asked respondents to self-report the degree to which they engage in uncivil communication on the web and then correlated that with trace data of participants’ social media content. The results indicated that those who self-disclose engaging in uncivil social media behavior also tend to generate uncivil content on social media, measured via the Google Perspective API. These dimensions of incivility have been tested across multiple domains and trained on substantial amounts of human-annotated comments [60].

**Table 1:** Incivility variable attributes.

Attribute name	Perspective API^a^ description [60]	Example post^b^
Severe toxicity	“A very hateful, aggressive, disrespectful comment or otherwise very likely to make a user leave a discussion or give up on sharing their perspective.”	“F*ck the vaccine and f*ck COVID, this should not be required period!!!”
Identity attack	“Negative or hateful comments targeting someone because of their identity.”	“DO NOT COMPLY. Screw liberals and their idiotic vaccine mandate.”
Insult	“Insulting, inflammatory, or negative comment towards a person or a group of people.”	“Bank accounts are frozen for protesting mandates. How many more vaccines will you take before you wisen up? Wake up you stupid little sheep.”
Profanity	“Swear words, curse words, or other obscene or profane language.”	“It must be hard to be a victim of the vaccine mandate. A**holes on the internet FROTH at the F*CKING mouth to dismiss your experience.”
Threat	“Describes an intention to inflict pain, injury, or violence against an individual or group.”	“I’ll put a bullet in someone who tries to force my kid to get the vaccine.”

^a^API: application programming interface.

^b^Curse words have been censored to make the table suitable for publication.

### Ethical Considerations

No personally identifiable information was included in this study. The institutional review board recognizes that the analysis of publicly available data does not constitute human subjects research. This study only used information in the public domain; thus, ethical review and approval was not required.

## Results

### Factor Analysis of Dimensions of Uncivil Discourse

Prior to hypothesis testing, we conducted a repeated measures ANOVA to assess whether to model dimensions of incivility together or separately. The main effect for the within-subjects factor was significant (*F*_4,32052_=930.44; *P*<.001), indicating significant differences among identity attack, insult, profanity, threat, and severe toxicity (see Table 2).

Tukey comparisons were used to test marginal mean differences in each combination of incivility dimensions. There were significant differences between each combination, except identity attack and profanity (see Table 3). Thus, we concluded that the 5 dimensions of incivility should be assessed separately in the main analysis.

**Table 2:** Means table for within-subject variables (N=8014).

Incivility dimension	Mean (SD)
Severe toxicity	0.10 (0.14)
Identity attack	0.12 (0.12)
Insult	0.18 (0.20)
Profanity	0.12 (0.18)
Threat	0.17 (0.15)

**Table 3:** The marginal means contrasts for each combination of within-subject variables for the repeated measures ANOVA.

Contrast	Difference	SE	*t* test (df)	*P* value
Severe toxicity – identity attack	–0.02	0.001	–15.11 (8013)	<.001
Severe toxicity – insult	–0.08	0.001	–66.07 (8013)	<.001
Severe toxicity – profanity	–0.02	0.0008	–25.79 (8013)	<.001
Severe toxicity – threat	–0.06	0.001	–43.18 (8013)	<.001
Identity attack – insult	–0.06	0.002	–36.78 (8013)	<.001
Identity attack – profanity	–0.004	0.002	–2.39 (8013)	.12
Identity attack – threat	–0.05	0.002	–30.34 (8013)	<.001
Insult – profanity	0.06	0.001	43.06 (8013)	<.001
Insult – threat	0.01	0.002	6.30 (8013)	<.001
Profanity – threat	–0.04	0.002	–21.48 (8013)	<.001

### Logistic Regression Analyses

#### Dichotomizing the Data

The skewed distribution of the data necessitated that we dichotomize the incivility dimensions for regression. The Google Perspective API recommends flagging a comment as having an attribute if it scores a 0.7 or higher—thus, this value was used to dichotomize the data for logistic regression [60]. Of the 8014 tweets, 53 (0.66%) contained identity attacks, 405 (5.05%) contained insults, 317 (3.96%) contained profanity, 137 (1.71%) contained threats, and 91 (1.14%) contained severe toxicity.

**Figure 2:**
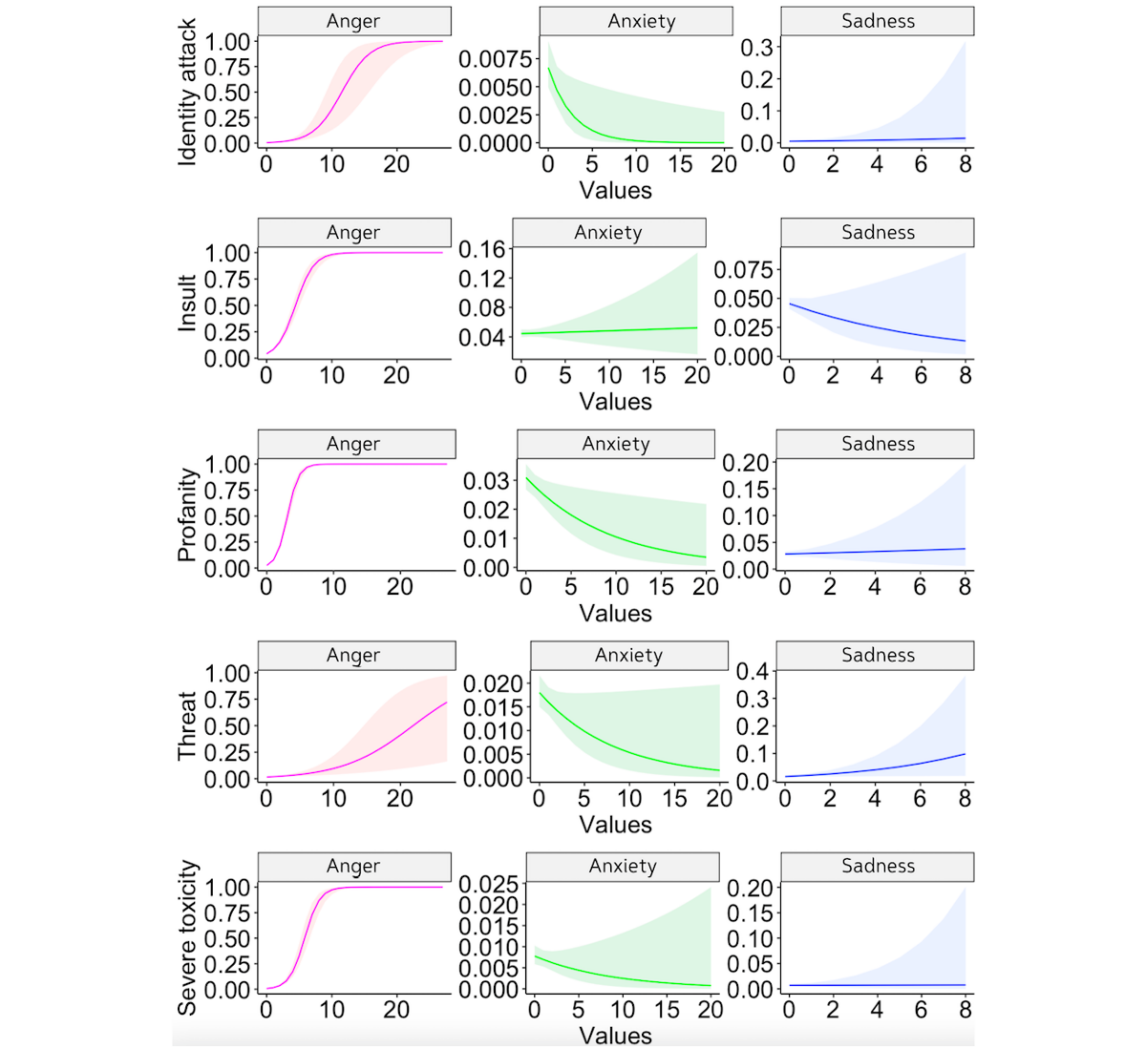
Negative emotion predicting the odds of severe toxicity, threat, profanity, insult, and identity attack. Scores for anger, anxiety, and sadness were computed using the Linguistic Inquiry and Word Count computerized coding tool that measures psychological processes in texts by counting the percentage of words in a given tweet that fall into prespecified categories.

For hypothesis testing, we conducted 5 logistic regression analyses to assess whether anger, anxiety, and sadness in posts predicted uncivil tweets (see Table 4 and Figure 2). Variance inflation factors for anxiety, sadness, and anger on all dimensions of incivility were less than 1.5, indicating there was not any multicollinearity between our independent variables.

**Table 4:** Binary logistic regression results with anxiety, anger, and sadness predicting dimensions of incivility. McFadden R2 was used to calculate model fit.

Variable		Odds ratio (95% CI)	*B*	*P* value	*R* ^2^		*χ* ^2^_3_	
**Threat**						.01		18.78
	(Intercept)		–4.04	<.001				
	Anxiety	0.88 (0.78-1.01)	–.12	.06				
	Sadness	1.27 (1.02-1.58)	.24	.04				
	Anger	1.21 (1.10-1.33)	.19	<.001				
**Identity attack**						.09		58.64
	(Intercept)		–5.06	<.001				
	Anxiety	0.70 (0.50-0.96)	–.36	.03				
	Sadness	1.15 (0.74-1.77)	.14	.54				
	Anger	1.59 (1.40-1.80)	.46	<.001				
**Profanity**						.22		567.15
	(Intercept)		–3.58	<.001				
	Anxiety	0.90 (0.81-0.98)	–.11	.02				
	Sadness	1.04 (0.83-1.31)	.04	.75				
	Anger	3.27 (2.93-3.67)	1.19	<.001				
**Insult**						.08		258.25
	(Intercept)		–3.13	<.001				
	Anxiety	1.01 (0.95-1.07)	.008	.79				
	Sadness	0.85 (0.67-1.10)	–.16	.22				
	Anger	2.03 (1.85-2.23)	.71	<.001				
**Severe toxicity**						.24		239.27
	(Intercept)		–.45	<.001				
	Anxiety	0.89 (0.75-1.06)	–.11	.20				
	Sadness	1.01 (0.65-1.57)	.01	.96				
	Anger	2.37 (2.12-2.66)	.86	<.001				

#### Anxiety

We found that the effect of anxiety on identity attack (*B*=–.36; odds ratio [OR] 0.70; *P*=.03) and profanity (*B*=–.11; OR 0.90; *P*=.02) were significant. However, contrary to our prediction that linguistic anxiety would increase incivility (H1), the results indicated that anxiety decreased the odds of identity attacks and profanity by approximately 30.48% and 10.43%, respectively. The results also reflected a stronger relationship between anxiety and identity attack than profanity. No other significant differences were found.

#### Anger

Consistent with our hypothesis (H2), the effect of anger on all 5 dimensions of incivility was significant (all *P*<.001). The results revealed that anger predicted the odds of profanity, insult, and severe toxicity to a greater extent than identity attacks and threats. The effect of the anger on threat (*B*=.19; OR 1.21; *P*<.001) and identity attack (*B*=.46; OR 1.59; *P*<.001) indicated that a 1-unit increase in anger increased the odds of threats by approximately 20.67% and identity attacks by approximately 58.9%. The effect of anger on insult (*B*=.71; OR 2.03; *P*<.001) and severe toxicity (*B*=.86; OR 2.37; *P*<.001) indicated that an increase in anger increased the odds of insults by approximately 103.15% and severe toxicity by approximately 137.29%. The results indicated that anger increased the odds of profanity the most (approximately 227.49%; *B*=1.19; OR 3.27; *P*<.001) when compared to the other 4 dimensions.

#### Sadness

H3 predicted that sadness will be positively associated with the level of incivility expressed in tweets. Our results showed that the effect of sadness on threat was significant (*B*=.24; OR 1.27; *P*=.04), indicating that a 1-unit increase in sadness increased the odds of threats by approximately 26.86%. Sadness did not have a significant effect on any other dimension of incivility.

## Discussion

### Principal Findings

Incivility has been understood as a multifaceted construct, encompassing the breadth of conceptual and operational definitions offered in the literature. This study resolved discrepant operationalizations of incivility by introducing incivility as a multifaceted construct and explored the distinct emotional processes underlying 5 dimensions of discourse incivility. The findings reveal that 3 types of emotions—anxiety, anger, and sadness—were significantly associated with dimensions of incivility. With regard to the relationship between anxiety and incivility, we found that the anxiety was negatively associated with identity attacks and profanity expressed in Twitter posts. Individuals who expressed higher levels of anger were more likely to engage in all 5 dimensions of incivility, including profanity, insults, severe toxicity, identity attacks, and threats. Lastly, our findings revealed that sadness was positively associated with uncivil behavior, especially threats.

### Comparison With Prior Work

Individuals who expressed higher anxiety were less likely to engage in uncivil behaviors such as posting hateful comments targeting individuals with a specific identity or using profane language in their posts. We suspect that individuals’ anxiety may have decreased the level of uncivil expressions about vaccine mandate policy, because individuals who are anxious about COVID-19 and its health consequences are more likely to seek ways to contain the threat (ie, spread of COVID-19) and exhibit positive attitudes and behaviors toward policies related to restricting the spread of COVID-19. Namely, when novel threatening stimuli are encountered and feelings of anxiety are induced, people may be motivated to attend to the issue at hand [66]. In line with this idea, previous studies suggest that anxiety can be an indicator of a “functional fear” that predicts individuals’ positive attitudes and behaviors (eg, compliance) toward COVID-19–related measures and policies [67]. For instance, an extant work shows that COVID-19–related anxiety and health-related fears were associated with more protective health behaviors and higher vaccine acceptance [68,69].

It is noteworthy that anger, unlike anxiety or sadness, predicted all dimensions of incivility, demonstrating that this emotion is the strongest predictor of incivility.

Evidence from previous studies has shown that prolonged risk and uncertainty about the level of risk can elicit anger and conflict within the community [70]. People have experienced increased levels of anger during the pandemic [71], and those who express anger have also exhibited disbelief toward COVID-19 vaccines [72]. Moreover, it has been shown that political polarization regarding the issues of vaccination and vaccine mandates has further fueled public outrage among groups with conflicting political views [51,52]. Thus, the strong association between anger and uncivil behaviors can be due to both social disruptions caused by the wide spread of COVID-19 and political conflicts partly induced by media outlets.

Lastly, as the level of sadness increased, individuals were more likely to exhibit verbal intentions to inflict pain and hurt other individuals or groups. Such aggression toward other people, especially exhibiting intentions to hurt others, may be explained by depression and victimhood. Approximately over 2 years of the COVID-19 pandemic, individuals worldwide have experienced prolonged social isolation and lifestyle disruptions, which have led them to be depressed [56,57]. Furthermore, the direct health impacts of the spread of COVID-19 have caused many individuals to become the victims of multiple losses such as a loss of financial security, loss of family members, and loss of physical/mental health and general safety [73,74]. However, sadness may have been strongly associated with viewing themselves as victims of COVID-19, which could have led them to issue threats to others who were favorable toward vaccine mandates. Additionally, this victimhood mentality [58] among the unvaccinated may have also been high because they are prohibited from participating in relationships and activities available to those who are vaccinated. This prohibition may have led them to feel socially excluded and in turn prompt threats toward the outgroup members—proponents of vaccine mandates.

### Limitations

Although the findings shed light on the psychological processes underlying vaccine mandate incivility, this study is not without limitations. The LIWC computational tool does not measure the nuances afforded by human coders. Although we endeavored to minimize this limitation by using well-validated measures [59], future work might employ human coders to analyze the specific topics related to uncivil discourse. Additionally, we focused on posts shared to Twitter and therefore cannot generalize our findings about incivility to other social media platforms. Given the role of platform community norms in predicting incivility, future work should investigate how incivility manifests itself on different platforms. Likewise, Twitter users are wealthier, younger, and more liberal than the wider population of Americans [75], and the sample was limited to English-speaking Twitter users, which makes it difficult to generalize our findings to the entire US population. Additionally, we acknowledge that social media posting data could have been biased based on individuals’ geographical area (eg, city and state), whether they were local residents or visitors in the area at the time of the post, as well as the types of activities completed during the course of a day [76,77]. These factors may have contributed to our study findings. Lastly, we did not measure how many different users were included in each stage in the data collection process. Future work should elucidate the extent to which a small number of active users produce uncivil vaccine mandate content.

### Conclusions

The results suggest that our multidimensional approach to incivility is a promising alternative to understanding and intervening in the psychological processes underlying uncivil vaccine discourse. Given the need for real-time monitoring and automated responses to the spread of health information and misinformation on the web, social media platforms can harness the Google Perspective API to offer users immediate, automated feedback when it detects that a comment is uncivil [78]. Furthermore, the Perspective API is available in 17 languages—from Arabic to Korean, enabling the study of uncivil health discourse in non-English posts. Future work should explore cross-cultural differences in uncivil health discourse.

**Figure 3:**
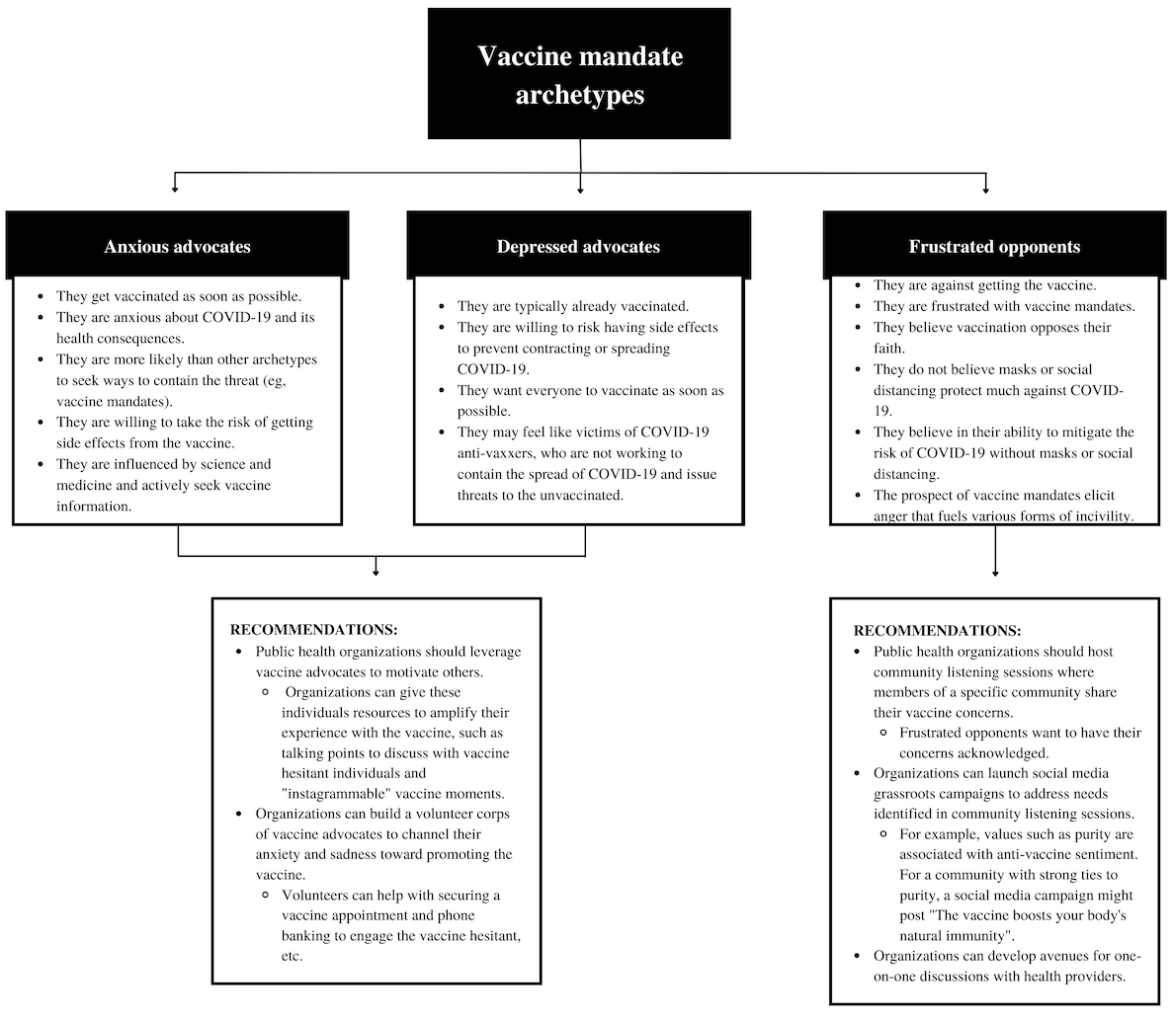
Concrete recommendations for promoting vaccine uptake based on underlying emotions.

Vaccine hesitancy still remains a threat to global health, and this work demonstrates that distinct emotional processes underlie distinct attitudes toward vaccines and vaccine-related policies. It is important for health practitioners and policy makers to first acknowledge negative emotions associated with vaccines and vaccine mandates while emphasizing the safety of COVID-19 vaccines in health campaigns, which would provide aid in reducing vaccine hesitancy. One avenue public health officials can take to combat vaccine hesitancy while simultaneously affirming discrete negative emotions toward the vaccine is by holding COVID-19 community listening sessions, where officials can hear directly from communities about COVID-19 concerns, including vaccination (see Figure 3 for an overview) [79]. After officials have a better understanding of the specific emotional processes underlying a communities’ vaccine hesitancy, public health campaigns can tailor messages to address these concerns (see Figure 3) [80,81].

## Abbreviations

API: application programming interface
H: hypothesis
LIWC: Linguistic Inquiry and Word Count
OR: odds ratio
